# A case of renal sarcoidosis complicated by parotid gland and uterine lesions

**DOI:** 10.1186/s12882-024-03635-6

**Published:** 2024-06-18

**Authors:** Shuhei Kitamoto, Mai Kaneko, Kyosuke Omata, Takashi Matsuyama, Hideo Yasuda, Ryuichi Furuya, Hirotaka Fukasawa

**Affiliations:** 1 Renal Division, Department of Internal Medicine, Iwata City Hospital, 512-3 Ohkubo, Iwata, Shizuoka 438-8550 Japan; 2 First Department of Medicine, Hamamatsu University School of Medicine, Hamamatsu, Shizuoka Japan

**Keywords:** Acute kidney injury, Parotid glands, Sarcoidosis, Uterus

## Abstract

**Background:**

Sarcoidosis is a systemic disease that can affect multiple organs. While pulmonary sarcoidosis is most commonly observed, renal sarcoidosis occurs less frequently. We herein report a case of sarcoidosis with an exceptionally rare distribution including renal lesions.

**Case presentation:**

A 51-year-old Japanese female was referred because of bilateral parotid swelling and renal dysfunction. Computed tomography scan showed the swelling of bilateral kidneys, parotid glands, and uterus. Ga scintigraphy also showed remarkable accumulation in these organs. Renal biopsy and cytological evaluations of parotid gland and uterus were performed and she was diagnosed as sarcoidosis of these organs. Treatment was initiated with prednisolone 40 mg/day and then renal dysfunction subsequently improved. In addition, the swelling of parotid glands and uterus improved and Ga accumulation in each organ had disappeared.

**Conclusion:**

This is a first case of renal sarcoidosis complicated by parotid glands and uterus lesions. Pathological findings and the reactivity observed in Ga scintigraphy indicated the presence of lesions in these organs.

## Background

Sarcoidosis is a multisystem disorder of unknown etiology characterized by the accumulation of T lymphocytes, mononuclear phagocytes and noncaseating granulomas in affected organs [1, 2]. In sarcoidosis, pulmonary involvement is the most prevalent, whereas the frequency of renal sarcoidosis remains unclear. In the previous study, 95% of 736 cases diagnosed with sarcoidosis showed pulmonary involvement, although only 0.7% cases showed renal sarcoidosis [3]. In addition, the prevalence of parotid gland sarcoidosis was 3.9% [3], and only a few cases have been reported in uterine sarcoidosis [4–6]. Herein, we report a first case of sarcoidosis in kidneys, parotid glands and uterus.

## Case presentation

**Fig. 1:**
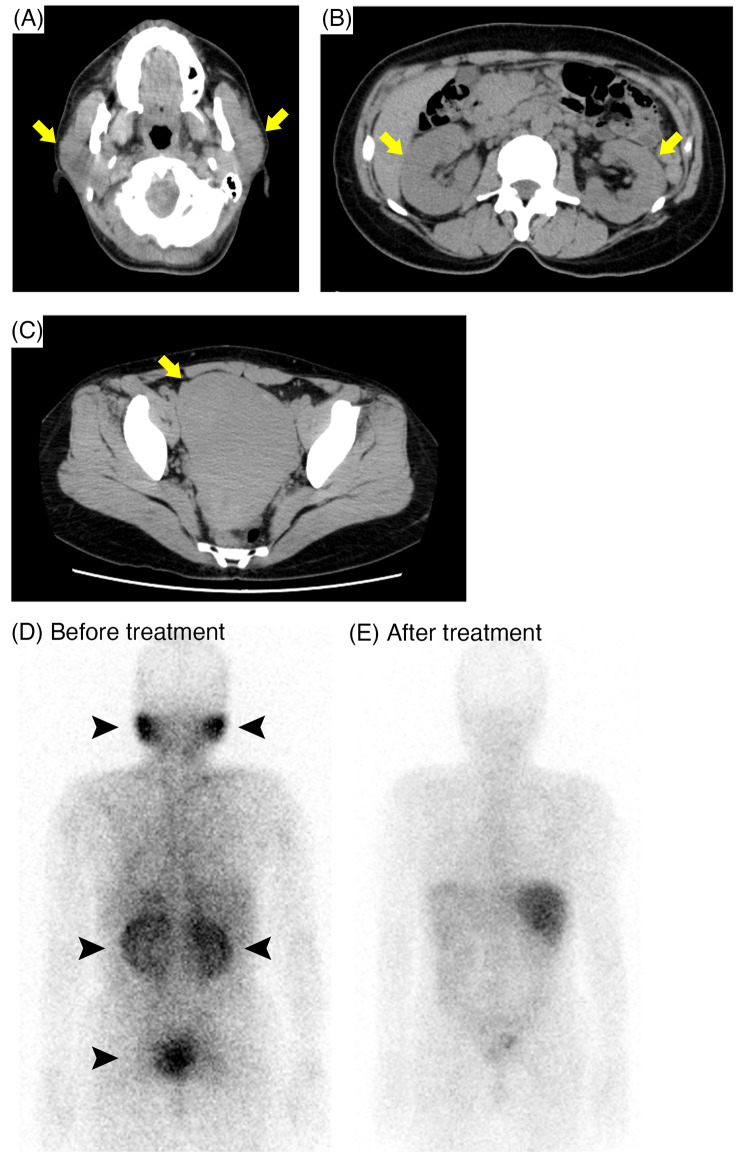
Findings of Computed tomography (CT) scan and Ga scintigraphy. (**A, B, C**) CT scan showed swelling of parotid glands (**A**), bilateral kidneys (**B**), and uterus (**C**, arrows). (**D**) Ga scintigraphy before treatment showed remarkable accumulation in these organs (arrowheads). (E) After the treatment, the accumulation has disappeared

A 51-year-old Japanese female was referred to our hospital with bilateral parotid swelling and renal dysfunction. There was no previous medical history of renal dysfunction. Physical examination revealed bilateral parotid swelling and lower legs edema. She also presented hypermenorrhea. Laboratory findings showed elevated levels of serum creatinine 3.1 mg/dL, serum corrected calcium 10.6 mg/dL, angiotensin converting enzyme (ACE) 44.9 U/L (reference range 8.3–21.4 U/L), and lysozyme 75 µg/mL (reference range 5.0–10.2 µg/mL). The urinary β2-microglobulin (β2-MG) level was also elevated (Table 1). Computed tomography (CT) scan revealed swelling of bilateral kidneys, parotid glands, and uterus (Fig. 1A, B, C, respectively). Additionally, Ga scintigraphy showed remarkable accumulation in these organs (Fig. 1D). Ocular screening was performed but uveitis was not indicated.

**Table 1:** Laboratory Data on admission

Complete blood count			Serology		
White blood cell	4,900	/µL	IgG	1,649	mg/dL
Red blood cell	403 × 10^4^	/µL	IgA	242	mg/dL
Hemoglobin	11.4	g/dL	IgM	170	mg/dL
Platelet	28.9 × 10^4^	/µL	IgG4	18.7	mg/dL
Blood chemistry			ANA	<×40	
Total protein	7.7	g/dL	MPO-ANCA	< 1.0	U/mL
Albumin	4.2	g/dL	PR3-ANCA	< 1.0	U/mL
ALP	66	U/L	ACE	44.9	U/L
AST	22	U/L	Lysozyme	75	µg/mL
ALT	16	U/L	25(OH)_2_Vit.D3	4.2	ng/mL
LDH	229	U/L	1–25(OH)_2_Vit.D3	135	pg/mL
γ-GTP	15	U/L	intact-PTH	9	pg/mL
BUN	33	mg/dL	PTHrP	< 1.0	pmol/L
Creatinine	3.1	mg/dL	sIL-2R	7,650	U/mL
eGFR	13		T-spot	(-)	
Uric acid	5.5	mg/dL			
Sodium	137	mEq/L	Urine analysis		
Potassium	4.1	mEq/L	pH	6.5	
Chloride	102	mEq/L	Protein	+	
Corrected calcium	10.6	mg/dL	Occult blood	±	
Phosphorus	3.8	mg/dL	Glucose	±	
CRP	0.34	mg/dL	β2-MG	101,202	µg/L
Amylase	167	U/L	NAG	12.8	U/L

Abbreviations: β2-MG, β2-microglobulin; γ-GTP, γ-glutamyl transpeptidase; ACE, angiotensin converting enzyme; ALP, alkaline phosphatase; ALT, alanine aminotransferase; ANA, anti-nuclear antibody; AST, aspartate aminotransferase; BUN, blood urea nitrogen; CRP, C-reactive protein; eGFR, estimated glomerular filtration rate; LDH, lactate dehydrogenase; MPO-ANCA, Myeloperoxidase-antineutrophil cytoplasmic antibody; NAG, N-cetylglucosaminidase; PR3-ANCA, Proteinase 3-antineutrophil cytoplasmic antibody; PTH, parathyroid hormone; PTHrP, parathyroid hormone related peptide; sIL-2R, soluble interleukin-2 receptor

**Fig. 2:**
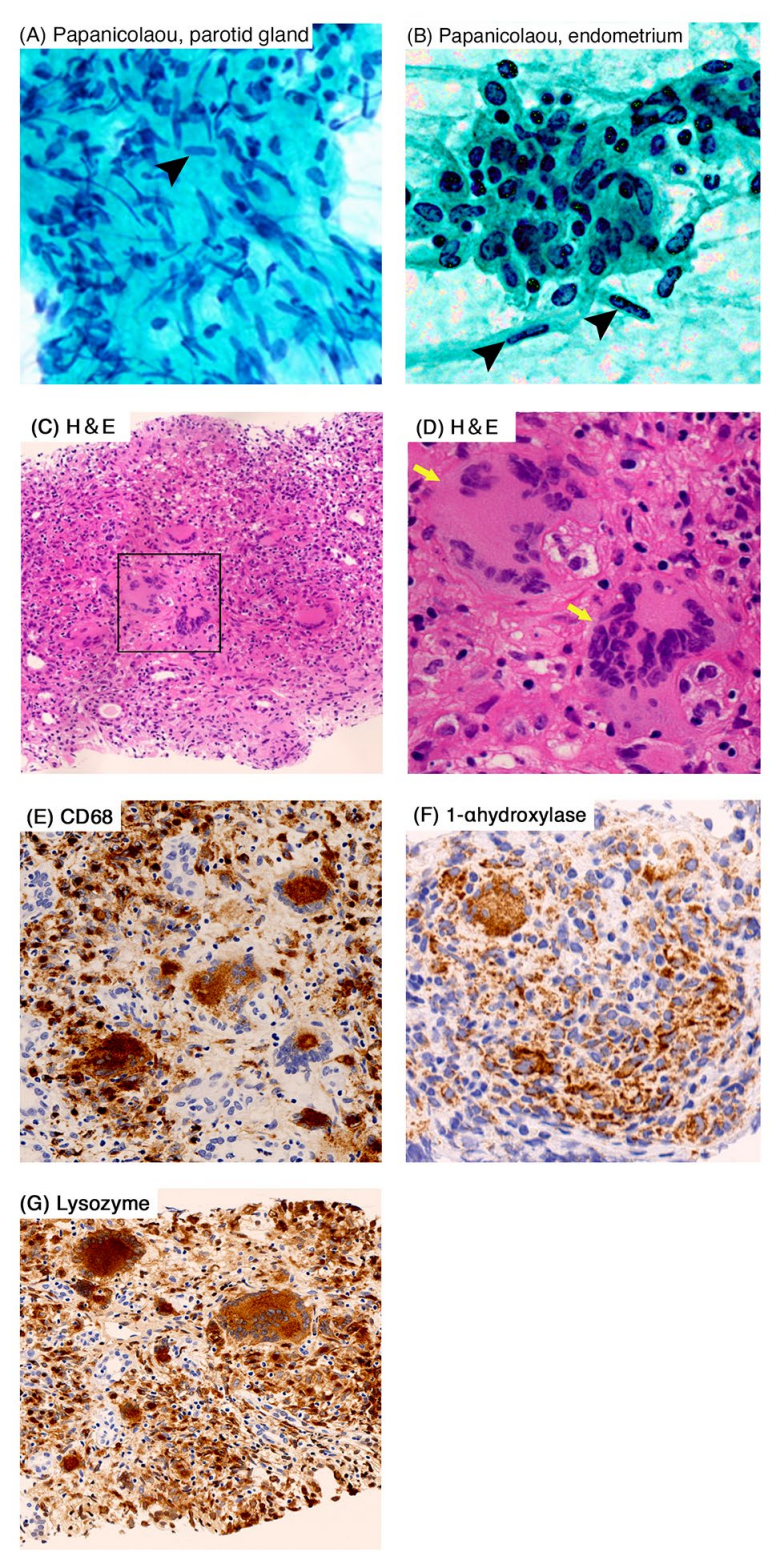
Cytologic findings of parotid gland and endometrium, and light microscopic findings of the renal biopsy. (**A** and **B**) Papanicolaou staining showed the presence of epithelioid cells in parotid gland (**A**) and endometrium (**B**). (**C** and **D**) Hematoxylin and eosin staining (**H&E**) showed the tubulointerstitial nephritis with diffuse non-caseating epithelioid cell granulomas and diffuse multinucleated giant cells (arrows). (**E**) Inflammatory cells and multinucleated giant cells showed positive in CD68 staining. (**F** and **G**) 1-α hydroxylase (**F**) and lysozyme staining (**G**) were positive in inflammatory cells and multinucleated giant cells. Original magnification, ×400 (**A, B, D**), x100 (**C**), ×200 (**E, F, G**), respectively

After admission, the needle aspiration cytology of parotid gland, endometrial cytology and renal biopsy were performed, respectively. Both parotid gland and endometrial cytology showed the presence of epithelioid cells indicating sarcoidosis (Fig. 2A, B, respectively). In the renal biopsy, light microscopy showed tubulointerstitial nephritis with diffuse non-caseating epithelioid cell granulomas and presence of diffuse multinucleated giant cells (Fig. 2C and D). A significant infiltration of inflammatory cells was observed in the interstitial area and around the renal tubules, with positive staining for CD68 (Fig. 2E), a specific marker of macrophages. Due to the presence of hypercalcemia and increased lysozyme level, we also performed 1-α hydroxylase (CYP27B1) and lysozyme staining. Both staining showed remarkable positivity in multinucleated giant cells and infiltrating cells (Fig. 2F, G, respectively). No notable findings were observed in the glomeruli, and all immune-fluorescence staining were negative.

**Fig. 3:**
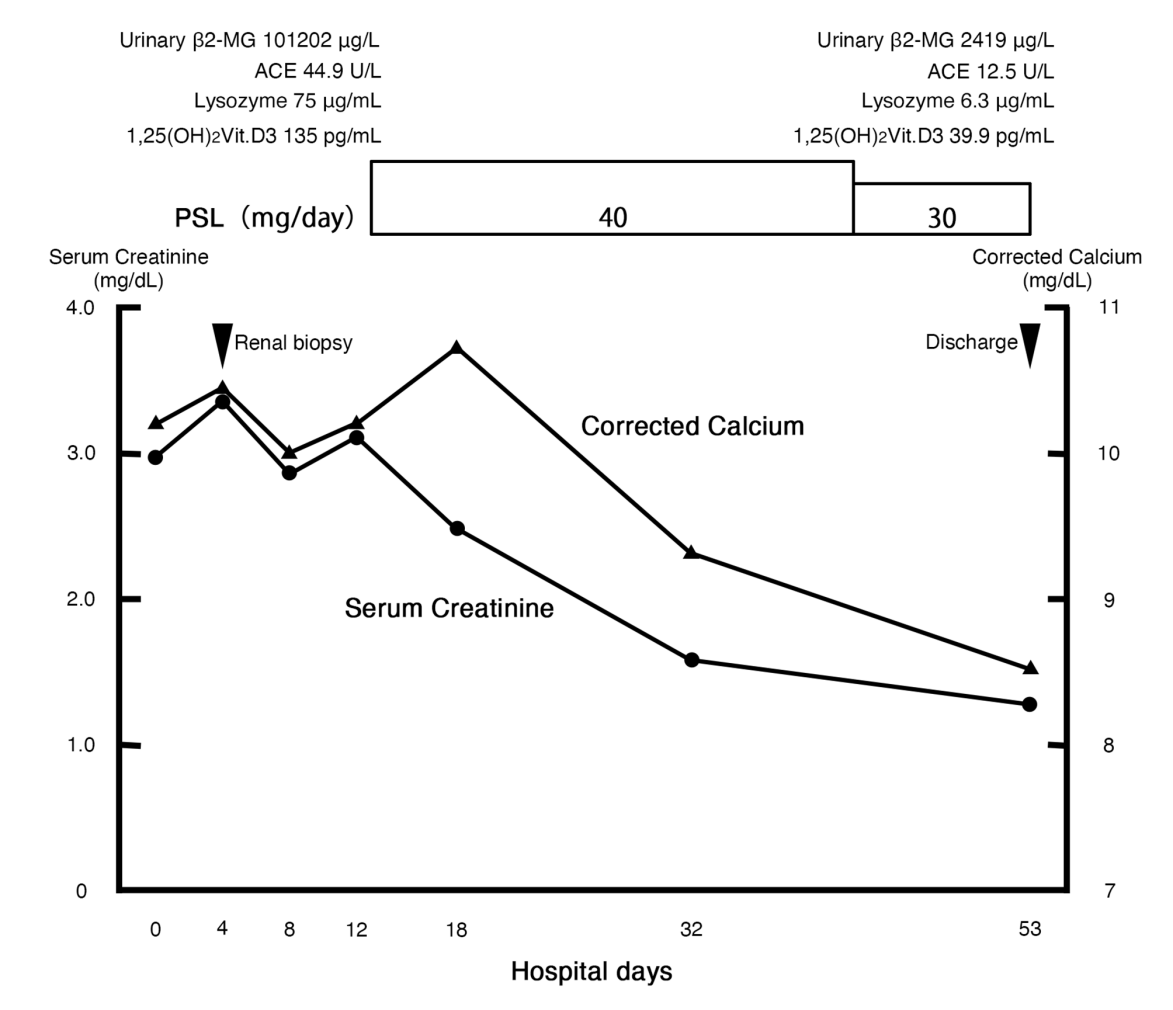
Clinical course after admission. ACE, angiotensin converting enzyme; β2-MG, β2-microglobulin; PSL, prednisolone

Based on the pathological findings, we diagnosed as renal sarcoidosis complicated by parotid glands and uterine lesions. As a treatment, prednisolone was initiated at a dose of 40 mg/day (0.8 mg/kg/day) since the 11th day, resulting in rapid improvement of renal function and hypercalcemia (Fig. 3). Furthermore, levels of urinary β2-MG, serum ACE, serum lysozyme, and serum 1,25(OH)_2_ vitamin D3 decreased. After the treatment, second Ga scintigraphy was performed, and the accumulation in the kidneys, parotid glands and uterus was disappeared (Fig. 1E). On the 53th day, the patient discharged.

After the discharge, the patient’s condition is stable without the apparent recurrence and serum ACE levels are also maintained within the normal range from 7.2 to 18.4 U/L for at least 30 months.

## Discussion

Sarcoidosis is a multisystem disorder of unknown etiology characterized by the infiltration of T lymphocytes and mononuclear phagocytes, and the formation of noncaseating granulomas in involved tissues [1, 2]. Sarcoidosis can affect a variety of organs, although pulmonary sarcoidosis is the most common organ involved. Baughman et al. [3]. reported that 95% cases exhibited pulmonary involvement, while only 0.9% cases exhibited renal involvement among 736 patients diagnosed as sarcoidosis. Parotid gland sarcoidosis has been reported to occur in approximately 5% of patients with sarcoidosis [3, 7]. On the other hand, there were only a few case reports in uterine sarcoidosis [4–6]. Therefore, the present case exhibited a very rare distribution of lesions as sarcoidosis. To the best of our knowledge, this is the first report of sarcoidosis in kidneys, parotid glands and uterus, although its relevance developed simultaneously in these organs is unknown.

In addition to its rarity of the distribution, several interesting findings were obtained in this case. First, inflammatory cells infiltrating into the renal tissue were positive for 1-α hydroxylase (CYP27B1) staining (Fig. 2F). Since these inflammatory cells were positive for CD68 (Fig. 2E), a specific marker for macrophages, these results indicated that 1-α hydroxylase might be originated from infiltrated macrophages into the renal tissue. In sarcoidosis, hypercalcemia has been reported to occur up to 10% cases and is caused by 1-α hydroxylase production from infiltrating macrophages into the affected organ resulting in the excessive formation of 1,25(OH)_2_ vitamin D3 [8]. This mechanism of hypercalcemia is primarily investigated in pulmonary sarcoidosis, and glucocorticoid therapy inhibits 1-α hydroxylase production in macrophages, leading to improvement of hypercalcemia [9, 10]. On the other hand, there are few reports investigating its mechanism of hypercalcemia in the other affected organs [11], while 1-α hydroxylase strongly stained infiltrating macrophages into the renal tissue and hypercalcemia was improved after glucocorticoid therapy in our case. Therefore, our case is highly suggestive to indicate that the same mechanism of hypercalcemia exists in renal sarcoidosis as that in pulmonary sarcoidosis. Second, lysozyme staining was positive in the renal tissue of this case. Sanada et al. [12] previously reported the significance of lysozyme staining in renal sarcoidosis. They actually performed lysozyme staining in various cases of tubulointerstitial nephritis from different etiologies and only cases of sarcoidosis consistently showed positive for lysozyme. Since lysozyme was also produced from infiltrating macrophages into the affected tissue of sarcoidosis same as 1-α hydroxylase [13, 14], the positivity of lysozyme staining could potentially serve as a novel supportive finding for the diagnosis of sarcoidosis. On the other hand, further studies should be needed to identify its diagnostic value.

In conclusions, we report a first case of renal sarcoidosis complicated by parotid glands and uterine lesions. The distribution of lesions in our case was exceptionally rare for sarcoidosis. Furthermore, both 1-α hydroxylase and lysozyme staining were positive for infiltrating cells, possibly macrophages in renal granulomatous lesions. 1-α hydroxylase in these cells could lead to hypercalcemia and positivity of lysozyme might contribute to a supporting finding for the diagnosis of sarcoidosis in the future.

## Data Availability

The datasets used and/or analyzed are available from the corresponding author upon reasonable request.

## Non-standard abbreviations

β2-MG: β2-microglobulin
γ-GTP: γ-glutamyl transpeptidase
ACE: angiotensin converting enzyme
ALT: alanine aminotransferase
ALP: alkaline phosphatase
ANA: anti-nuclear antibody
AST: aspartate aminotransferase
BUN: blood urea nitrogen
eGFR: estimated glomerular filtration rate
LDH: lactate dehydrogenase
MPO-ANCA: Myeloperoxidase-antineutrophil cytoplasmic antibody
NAG: N-cetylglucosaminidase
PR3-ANCA: Proteinase 3-antineutrophil cytoplasmic antibody
PTH: parathyroid hormone
PTHrP: parathyroid hormone related peptide
sIL-2R: soluble interleukin-2 receptor
